# Renal Regenerative Potential of Extracellular Vesicles Derived from miRNA-Engineered Mesenchymal Stromal Cells

**DOI:** 10.3390/ijms20102381

**Published:** 2019-05-14

**Authors:** Marta Tapparo, Stefania Bruno, Federica Collino, Gabriele Togliatto, Maria Chiara Deregibus, Paolo Provero, Sicheng Wen, Peter J. Quesenberry, Giovanni Camussi

**Affiliations:** 1 Department of Medical Sciences and Molecular Biotechnology Center, University of Torino, 10126 Torino, Italy; marta.tapparo@unito.it (M.T.); stefania.bruno@unito.it (S.B.); gabriele.togliatto@unito.it (G.T.); 2 Department of Biomedical Sciences and Paediatric Research Institute “Citta della Speranza”, University of Padova, 35129 Padova, Italy; federica.collino@unipd.it; 3 2i3T Società per la gestione dell’incubatore di imprese e per il trasferimento tecnologico Scarl, University of Torino, 10126 Torino, Italy; mariachiara.deregibus@unito.it; 4 Department of Molecular Biotechnology and Health Sciences and Molecular Biotechnology Center, University of Torino, 10126 Torino, Italy; paolo.provero@unito.it; 5 Division of Hematology/Oncology, Brown University, Rhode Island Hospital, Providence, Rhode Island, RI 02912, USA; swen@lifespan.org (S.W.); PQuesenberry@lifespan.org (P.J.Q.)

**Keywords:** mesenchymal stromal cells, extracellular vesicles, acute kidney injury, modified-MSCs, microRNA

## Abstract

Extracellular vesicles (EVs) derived from mesenchymal stromal cells (MSCs) possess pro-regenerative potential in different animal models with renal injury. EVs contain different molecules, including proteins, lipids and nucleic acids. Among the shuttled molecules, miRNAs have a relevant role in the pro-regenerative effects of EVs and are a promising target for therapeutic interventions. The aim of this study was to increase the content of specific miRNAs in EVs that are known to be involved in the pro-regenerative effect of EVs, and to assess the capacity of modified EVs to contribute to renal regeneration in in vivo models with acute kidney injuries. To this purpose, MSCs were transiently transfected with specific miRNA mimics by electroporation. Molecular analyses showed that, after transfection, MSCs and derived EVs were efficiently enriched in the selected miRNAs. In vitro and in vivo experiments indicated that EVs engineered with miRNAs maintained their pro-regenerative effects. Of relevance, engineered EVs were more effective than EVs derived from naïve MSCs when used at suboptimal doses. This suggests the potential use of a low amount of EVs (82.5 × 10^6^) to obtain the renal regenerative effect.

## 1. Introduction

Mesenchymal stromal cells (MSCs) are one of the most studied adult stem cells and have been extensively applied in the field of regenerative medicine. In the last years, many studies have demonstrated that their therapeutic effects are mainly mediated by the secretion of bioactive molecules (such as RNAs, proteins, and lipids) that can be directly released in the local microenvironment or packaged in extracellular vesicles (EVs) [1]. Paracrine factors released by MSCs, including EVs, induce the recovery of injured tissue and modulate the immune response and inflammation [2,3]. Many reports have demonstrated the application of MSCs and of their derived EVs in the recovery of renal dysfunction [4]. Different preclinical models of acute and chronic kidney injuries have been used to demonstrate the efficacy of EVs from MSCs in the amelioration of acute kidney injury (AKI) and in preventing progression at the chronic stage [5]. Recently, we tested different subpopulations of MSC-derived EVs in renal regeneration. Most of the effects observed in the recovery from AKI were ascribed to the exosomal fraction, which carried mRNA, miRNAs and proteins that induced the proliferation of tubular cells. Despite the inefficiency of the non-exosomal fraction, we observed that the effect of the exosomal fraction and the total-EVs was not significantly different in terms of pro-regenerative potential, suggesting that the ineffective fraction did not interfere with the exosomal fraction activity [6]. Moreover, MSCs may be manipulated in culture by transferring specific miRNA to EV-producing cells to obtain modified-EVs with potentiated pro-regenerative or reparative effects [7,8,9,10,11]. In this work, we set up a method to increase—in MSCs and in their total-EVs—the content of specific miRNAs involved in renal regeneration. To assess the capacities of modified-EVs to contribute to AKI recovery, we tested them in vitro on murine renal tubular epithelial cells and in vivo in a model of AKI induced by glycerol injection.

## 2. Results

### 2.1. Identification of Pro-Regenerative miRNAs Carried by EVs

In our previous study, we performed RNA sequencing (RNA-Seq) analysis to detect the molecular changes that occurred in the kidneys of AKI mice treated with MSC-derived EVs (EV-CTRL) vs. untreated AKI mice (AKI) (Gene Expression Omnibus accession number GSE59958) [12]. In this study, the potential healing miRNAs were selected from among known human miRNAs that were predicted to significantly down-regulate RNAs modulated in our treatments with a bio-informatic approach, as described in the Methods section. The obtained miRNA families (Table 1) were posteriorly cross-matched with a list of 50 miRNAs with increased expression in the MSC-derived EVs [6]. The following miRNAs were identified: miR-10a-5p, miR-127-3p, miR-29a-3p, let-7a and miR-30a-5p. miR-486-5p was also selected because it was the most enriched miRNA in the exosomal fraction, which was the more effective fraction in promoting AKI recovery [6].

**Figure 1:**
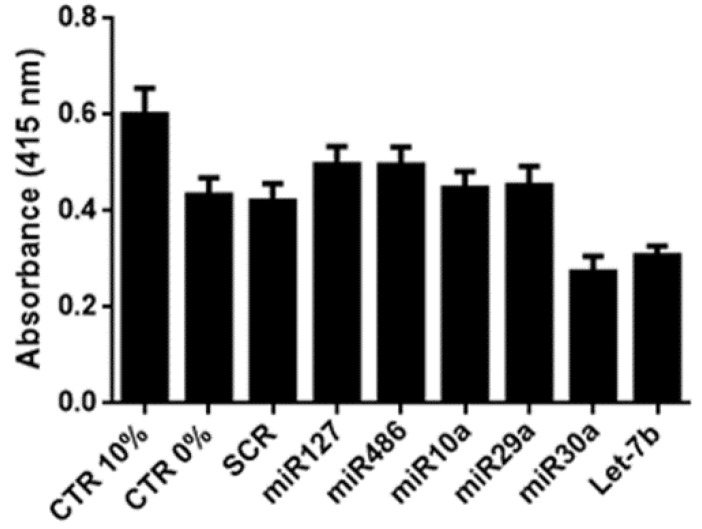
In vitro effect of selected miRNA mimics. Different miRNA mimics (miR127, miR486, miR10a, miR29a, miR30a and let-7b-100 nM) were added to murine tubular epithelial cells (mTECs) maintained in hypoxia for 48 h, after which proliferation was evaluated following 24 h of re-oxygenation. mTECs were maintained in medium supplemented with 10% fetal calf serum (FCS) or 0% FCS, used respectively as the positive (CTR 10%) and negative (CTR 0%) controls. Data are reported as mean ± SEM for three different experiments performed in quadruplicate.

To evaluate the potential of selected miRNAs to promote tubular cell proliferation, murine tubular epithelial cells (mTECs) were treated with miRNA mimics and submitted to hypoxia/reoxygenation conditions. mTECs treated with miR-10a-5p (miR10a), miR-127-3p (miR127), miR-29a-3p (miR29a) and miR-486-5p (miR486) were able to proliferate to some degree in the hypoxia/reoxygenation culture, even if the obtained results did not reach statistical significance, unlike let-7b- and miR-30a-5p- (miR30a-)treated tubular cells (Figure 1), which only supported the beneficial effects of selected EV-miRNAs in AKI.

### 2.2. Generation of MSCs and Derived EVs Engineered with miRNAs

**Figure 2:**
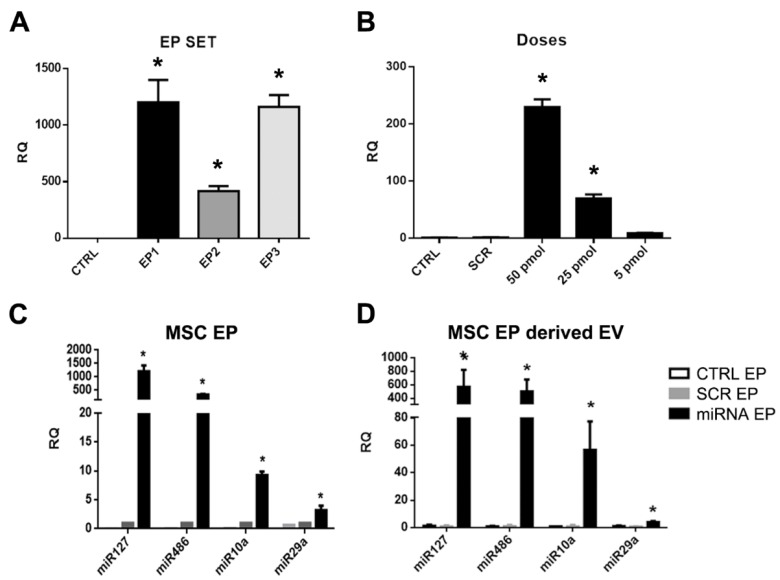
miRNA enrichment evaluation in mesenchymal stromal cells subjected to electroporation (MSC-EP) and derived EVs. (**A**) Representative real-time PCR showing the efficiency of transfection for different electroporation protocols (EP1-3). ANOVA with Dunnett’s multiple comparison test was performed. * *p* < 0.05 EP protocol vs. CTRL (non elecroporated cells); (**B**) representative real-time PCR showing the efficiency of transfection for different doses of control miRNA mimic obtained with EP1 electroporation conditions. ANOVA with Dunnett’s multiple comparison test was performed. *p* < 0.05 different control miRNA doses vs. scramble (SCR); (**C**) representative real-time PCR showing the increased expression of the selected miRNAs (miR10a, miR29a, miR127, miR486) 24 h after transfection in MSC-EP and (**D**) in derived EVs collected 24 h after transfection. Data were normalized in respect to MSC transfected with the SCR. Data are represented as relative quantification (RQ) ± SEM. * *p* < 0.05 MSC-EP SCR vs. MSC-EP transfected with mimics and * *p* < 0.05 EV-SCR vs. EV transfected with mimics. Multiple *t* test with Holm–Sidak method correction was performed.

MSCs were subjected to electroporation (MSC-EP) in order to enrich their content of the selected miRNAs. Different electroporation protocols were tested (EP1-3, Table 2, Materials and Methods section) and transfection efficiency was evaluated by qRT-PCR. The EP1 protocol (990 V, 40 msec, 1 pulse) was selected because of the enhanced expression of the control miRNA mimic observed in the MSCs and the maintained cell viability (Figure 2A). We also set up the optimal dose of miRNA mimic to transfect MSCs. As seen in Figure 2B, 25 pmol/4 × 10^4^ cells was the lowest dose tested that increased the expression of the control miRNA. Expression of the selected miRNAs was then assessed using the transfection condition identified during setting. We detected an increased expression of the selected miRNAs in MSC-EP after 24 h (Figure 2C) that remained stable until day 7 (not shown). Of notice, we observed a higher expression for miR-127 and miR-486 with respect to the other two miRNAs, suggesting a different efficiency of transfection.

**Figure 3:**
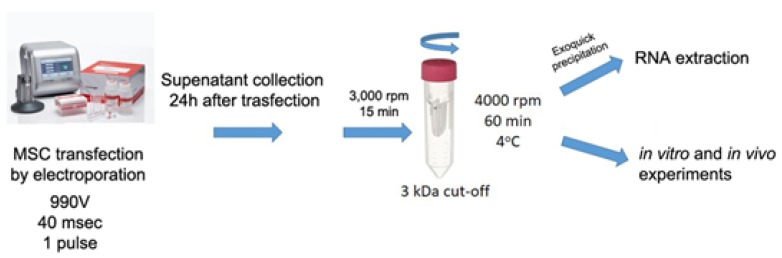
Schematic illustration of the engineering method used to transiently transfect MSCs. MSCs were transfected via a neon electroporation system with different miRNA mimics. EVs were collected after 24 h and supernatant, previously centrifuged at 2000 *g* to eliminate cell debris, was concentrated by 3 kDa filter tube. EVs were then used for in vitro and in vivo experiments or precipitated by Exoquick and used for RNA extraction.

**Figure 4:**
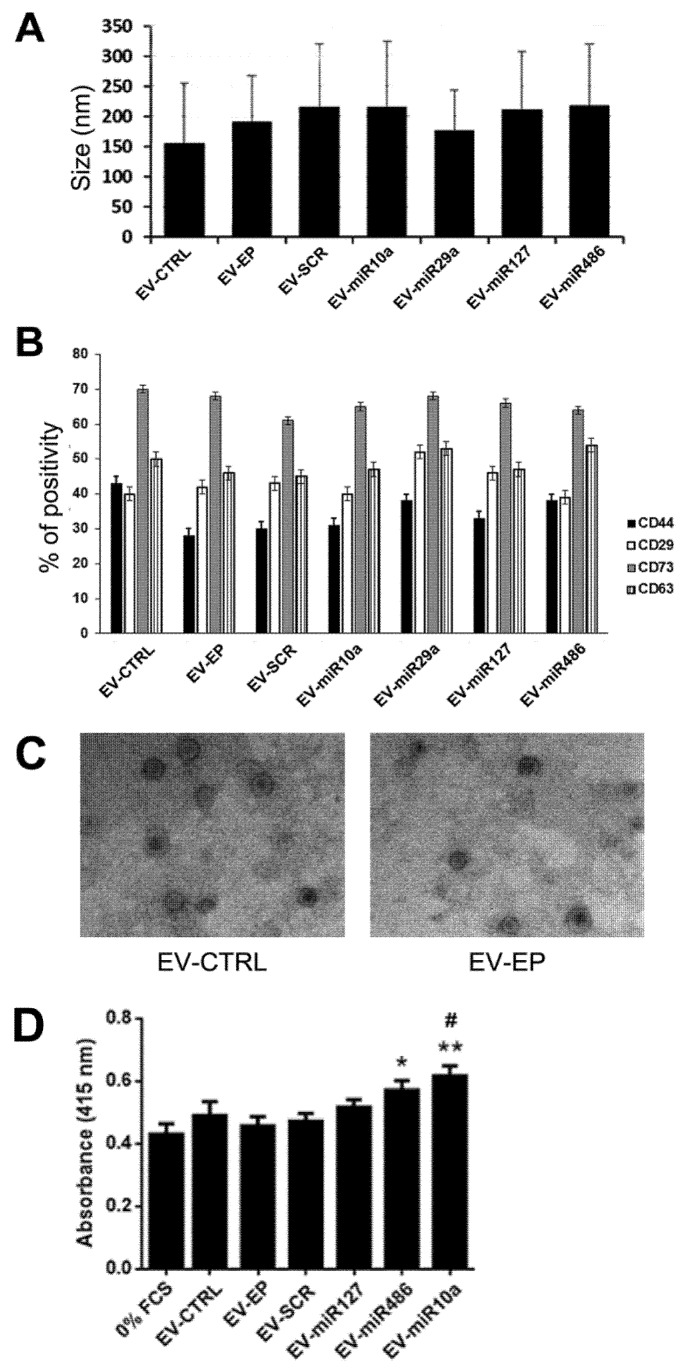
Characterization of EVs derived from naïve MSCs and from EP MSCs. (**A**) Size of EVs obtained from naïve MSC (EV-CTRL), from electroporated MSC (EV-EP) and transfected with scramble (EV-SCR) or with different miRNA mimics (EV-mir10a, EV-miR29a, EV-miR127, EV-miR486), evaluated by nanoparticle tracking analysis (NTA). Data reported are mean ± SD for three different experiments. No statistically significant differences were observed among the different types of EVs; (**B**) cytofluorimetric analysis of the expression of MSC (CD44, CD29 and CD73) and exosomal (CD63) markers, in different EV populations. Data reported are the mean ± SD for three different experiments. No statistically significant differences were observed in marker expression among the different types of EVs; (**C**) representative micrographs of transmission electron microscopy of EV-CTRL (left) and EV-EP (right). EVs negatively stained with NanoVan (magnification ×100,000); and (**D**) different types of EVs (1000/cells) were added to mTECs maintained for 48 h in hypoxia, after which proliferation was evaluated following 24 h of reoxygenation. Data are reported as mean ± SEM for three different experiments in quadruplicate. ANOVA with Dunnett’s multiple comparison test was performed. ** *p* < 0.01 and * *p* < 0.05 mTEC stimulated with EV-mimic vs. mTEC maintained in 0% FCS, ^#^ *p* < 0.05 EV-miR10a vs. mTEC stimulated with EV-EP.

EVs from MSC-EP (EV-EP), enriched with different miRNA mimics (Figure 3), were isolated 24 h after transfection and subsequently characterized for size distribution and particle number by NanoSight. EVs derived from MSC-EP showed similar size profiles to naïve MSC EVs. No differences were observed among EV-EP that were simply electroporated or transfected with scramble (SCR) mimic (EV-SCR) or with different miRNAs mimics (EV-miR127, EV-miR486, EV-miR10a, EV-miR29a) (Figure 4A). Moreover, evaluation of particle number did not show any variation between the different mimic-enriched EV-EP (not shown).

Additionally, typical MSC marker expression on EV-EP was maintained, suggesting that electroporation did not affect surface molecule expression. As described in Reference [6], EVs derived from naïve MSCs or MSC-EP in the presence of different miRNA mimics expressed the typical exosomal marker (CD63) and MSC markers (CD73, CD44 and CD29) (Figure 4B). Electron microscopy confirmed that EV-EP maintained the same morphology as EV-CTRL (Figure 4C).

miRNA enrichment was then evaluated by real-time PCR for the different EV-EP. The expression of miR-127, miR-10a, miR-29a and miR-486 was increased in MSC-EP-derived EVs with respect to MSC-EVs derived from cells that were only electroporated or from SCR-transfected MSC-EP (Figure 2D). As seen in the MSC-EP and their respective EVs, the efficiency of enrichment was slightly different according to the mimic used. For instance, miR-29a was less enriched in EVs with respect to the other mimics. Instead, we observed a higher increase in the expression for miR-127 and miR-486.

### 2.3. In Vitro and In Vivo Effects of EVs

EVs obtained from naïve MSCs and from MSC-EP transfected with SCR or with 10a, 127 and 486 miRNA mimics were tested in vitro on mTECs cultured in hypoxia. EV-miR29a was not further tested since the miR-29a in EVs after transfection was less enriched in comparison with the other miRNAs considered. EVs from MSC-EP transfected with 10a and 486 miRNA mimics induced significant proliferation of mTECs with respect to the negative controls (mTECs maintained with 0% fetal calf serum [FCS]). Only EVs enriched with miR10a induced a significant increase in proliferation with respect to EVs obtained by MSC-EP (Figure 4D).

**Figure 5:**
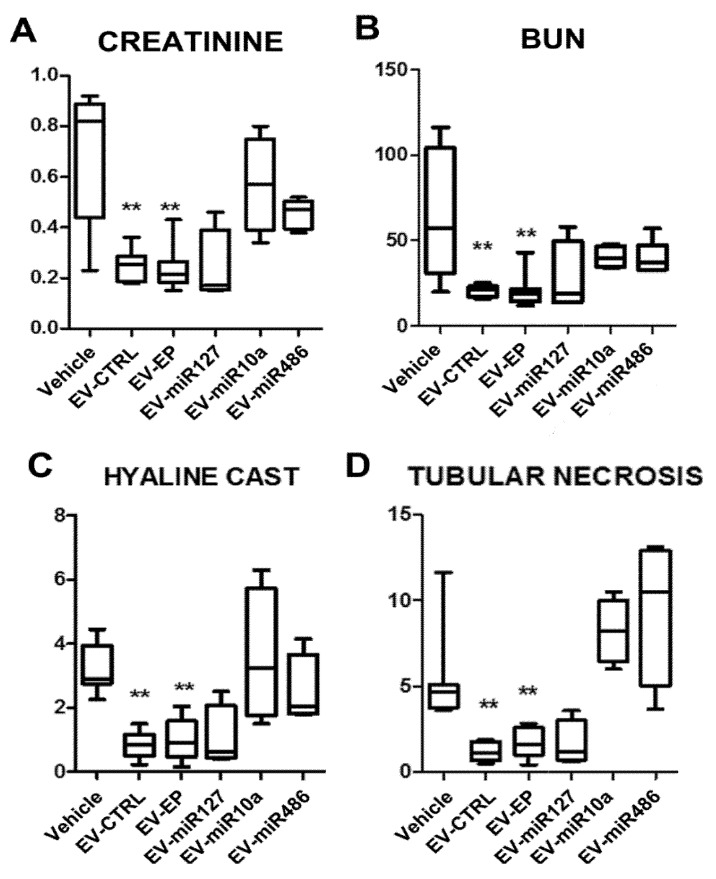
Effect of EVs derived from naïve or electroporated MSC in AKI mice: Functional and morphological evaluation. (**A**) Creatinine and (**B**) blood urea nitrogen (BUN) values in AKI mice injected with vehicle alone (Vehicle) or with 165 × 10^6^ EVs derived from naïve MSCs (EV-CTRL) or from electroporated MSCs (EV-EP) transfected with different miRNA mimics (EV-miR127, EV-miR10a, EV-miR486). EVs were injected at day 3 and mice were sacrificed at day 5 after glycerol administration. Results are expressed as mean ± SD; ANOVA with Dunnett’s multiple comparison test was performed. ** *p* < 0.01 EV-CTRL and EV-EP vs. Vehicle. Morphometric evaluation of (**C**) hyaline casts and (**D**) tubular necrosis in AKI mice treated with vehicle alone (Vehicle) or injected with 165 × 10^6^ EVs derived from naïve MSCs (EV-CTRL) or from electroporated MSCs (EV-EP) transfected with different miRNA mimics (EV-miR127, EV-miR10a, EV-miR486). Results are expressed as mean ± SD; ANOVA with Dunnett’s multiple comparison test was performed. ** *p* < 0.01 EV-CTRL and EV-EP vs. Vehicle.

MSC electroporation did not interfere with the renal pro-regenerative capacity of EVs; EV-EP significantly improved renal function and morphology similarly to naïve MSC-EVs (Figure 5).

Treatment of AKI mice with a dose of miRNA-enriched EVs comparable to the effective dose of EV-CTRL (165 × 10^6^ EV/mouse) did not show any significant improvement. In fact, in some experimental conditions (EV-miR10a and EV-miR486), a worsening was even observed, suggesting that changing the composition of miRNAs content in MSC-EVs altered their pro-regenerative properties (Figure 5). The administration of EV-miR127 did not show a worsening of kidney function and morphology, nor an amelioration of kidney function (Figure 5).

**Figure 6:**
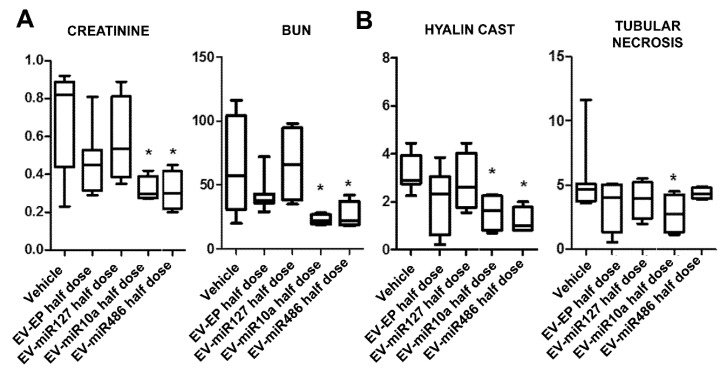
Effect of EVs derived from naïve or miRNA-enriched MSC in AKI mice: Functional and morphological evaluation. (**A**) Creatinine and BUN values in AKI mice injected with vehicle alone (Vehicle) or with 82.5 × 10^6^ EVs derived from electroporated MSCs (EV-EP) and transfected with different miRNA mimics (EV-miR127, EV-miR10a, EV-miR486). EVs were injected at day 3 and mice were sacrificed at day 5 after glycerol administration. Results are expressed as mean ± SD; ANOVA with Dunnett’s multiple comparison test was performed. * *p* < 0.05 EV-miR10a half-dose and EV-miR486 half-dose vs. Vehicle. (**B**) Morphometric evaluation of hyaline casts and tubular necrosis in AKI mice treated with vehicle alone (Vehicle) or with 82.5 × 10^6^ EVs derived from electroporated MSCs (EV-EP) and transfected with different miRNA mimics (EV-miR127, EV-miR10a, EV-miR486). Results are expressed as mean ± SD; ANOVA with Dunnett’s multiple comparison test was performed. * *p* < 0.05 EV-miR10a half-dose and EV-miR486 half-dose vs. Vehicle for hyaline cast; * *p* < 0.05 EV-miR10a half-dose vs. Vehicle, for tubular necrosis.

To better understand whether miRNA-enriched EVs could improve the biological activities of EVs, we treated AKI mice with an ineffective dose of unmodified EVs. A half-dose of these EVs (82.5 × 10^6^ EV/mouse) did not induce significant functional or morphological improvements (Figure 6). In contrast, half-doses of EVs obtained from MSC-EP transfected with miRNA mimic-486 or -10a induced a significant amelioration of renal function and morphology (Figure 6). These findings suggest that the regenerative effect of MSC-derived EVs is related to a balanced composition of miRNAs and, therefore, modification in miRNA content may increase the effectiveness of EVs at lower doses, but not improve the effective dose of naïve EVs.

## 3. Discussion

Recent studies have shown that paracrine mechanisms, including EVs, are responsible for stem/progenitor cell-mediated renal regenerative effect [5]. EVs derived from MSCs shuttle different molecules (proteins, lipids, and nucleic acids) that may contribute to their pro-regenerative potential. Among the different shuttled molecules, miRNAs have been reported to be one of the factors involved in the pro-regenerative effects of MSC-EVs. Indeed, miRNA deregulation by Drosha-knockdown in MSCs has been reported to inhibit the regenerative potential of MSCs and of their derived EVs in a murine model of AKI [12]. This suggests a critical role of miRNA content in MSCs and MSC-EVs in the recovery following an AKI.

The idea of using EVs as carriers for selected miRNA cargo became very attractive in the last decades. EVs overcome many problems related to stability and preservation of their cargo from degradation in circulation. Different approaches were developed in order to modify EV content to enhance their homing capacity (proteins) or their effect (miRNAs, mRNAs and drugs) [13]. Direct manipulation of EVs implies the temporary disruption of EV membranes by different techniques, such as electroporation, sonication or chemical transfection [14]. Another technique used to enhance miRNA content in EVs is engineering of the parental cells. Some recent works demonstrated that is possible to load miRNA mimic or antimiR into MSCs by transfection, as well as to increase the pro-regenerative properties of EVs in different tissue injuries [7,8,9,10,11,15] or to potentiate their anti-tumor effect [16,17].

In this study, we set up a method to increase the content of specific miRNAs involved in renal regeneration in MSCs and their respective EVs. Potentially regenerative miRNAs were selected using a bio-informatic approach based on predicted interactions between the miRNAs present inside the MSC-EVs with genes modulated during AKI treatment with MSC-EVs. Moreover, this list was implemented with miR-486-5p, which was highly expressed in the exosomal fraction of MSC-EVs [6]. Of relevance, miR-486-5p and some of the miRNAs selected by bio-informatic analyses (miR-10a-5p, miR-29a-3p) were found to be down-regulated in EVs obtained by Drosha knock-down MSCs, which were ineffective in glycerol induced AKI [12].

In this work, we demonstrated that the miRNA mimics transfected in MSCs were also up-regulated in their EVs. Among the different miRNA mimics transfected, miR-127 and miR-486 were more enriched than miR-29a in cells and, consequently, in their EVs, suggesting differences in efficiency of transfection for different miRNAs.

The EVs obtained from transfected cells with miR-10a, -127 and -486 were tested in vivo. Treatment with a dose of miRNA-enriched EVs known to be effective in the AKI model [6] did not provide any significant improvement, while a worsening was observed for miR-10a- and miR-486-enriched EVs. These data suggest that changing the miRNA composition of MSC-EVs can alter their renal regenerative capacities. When we used an ineffective dose of naïve-EVs as the control, and a similar dose miR-10a- and miR-486-enriched EVs, a significant improvement of renal function and morphology was invariably observed. These findings indicate that in order to detect biological activity of miRNA-enriched EVs, low doses of EV should be studied. Moreover, these data highlight the important role of miR-10a and of miR-486 in the pro-regenerative effect exerted by MSC-EVs in AKI. Interestingly, miR-486 is also in exosomes derived from human endothelial progenitor cells, which have been shown to possess renal regenerative capacity in ischemia-reperfusion injury models [18].

In conclusion, our study has demonstrated that it is possible to modify the miRNA content of MSCs and their EVs. Changing the amount of pro-regenerative miRNAs in EVs was found to modify the window of biological activity of these EVs. Therefore, this may be a strategy to reduce the amount of EVs used in therapy. We previously showed that EVs derived from MSCs accumulated specifically in the kidneys of mice with AKI compared to healthy controls [19]. Since in the present study the bio-distribution of miRNA-enriched EVs was not evaluated, we cannot rule out the possibility that miRNA-enriched EVs have a different bio-distribution.

## 4. Materials and Methods

### 4.1. Cell Cultures and EV-CTRL Isolation

Bone marrow MSCs were purchased from Lonza (Basel, Switzerland) and cultured in a mesenchymal stem cells basal medium bullet kit (Lonza). MSCs were used for the different experiments until passage 6 and expressed the typical MSC markers (CD105, CD29, CD73, CD44 and CD90) (not shown).

MSC-EVs, used as control (EV-CTRL), were obtained by ultracentrifugation, as described in Reference [6]. Briefly, EVs were obtained from supernatants of MSCs cultured overnight in (Roswell Park Memorial Institute (RPMI) medium. After removal of cell debris and apoptotic bodies by centrifugation at 3000× *g* for 20 min, EVs were purified by 2 h ultracentrifugation at 100,000× *g* at 4 °C. EVs from control MSCs or from modified MSCs were used freshly or stored at −80 °C after resuspension in RPMI supplemented with 1% dimethyl sulfoxide (DMSO, Sigma, St. Louis, MO, USA).

Murine renal tubular epithelial cells (mTECs) were obtained as previously described [20] and cultured in Dulbecco’s Modified Eagle Medium (DMEM) low glucose (Euroclone, Pero, Italy) supplemented with 10% fetal calf serum (FCS, Euroclone), penicillin (50 IU/mL), and streptomycin (50 µg/mL) (Sigma). Murine TECs were characterized for positive staining to cytokeratin, alkaline phosphatase and aminopeptidase A, and for negative staining for endothelial (von Willebrand factor), hematopoietic (CD45) and glomerular (nephrin) markers.

### 4.2. MSC Transfection and Collection of Engineered MSC-EVs

To obtain miRNA-enriched MSCs, cells were transiently transfected by electroporation (MSC-EP) (neon transfection system) using a 100 µl tip (Thermo Fisher Scientific, Waltham, MA, USA), according to the manufacturer’s protocol. Different electroporation conditions with a control miRNA mimic (100 nM) were tested in order to find the optimal protocol (Table 2 and Figure 2A). The electroporation conditions were set to 990 V, 40 msec and 1 pulse (EP1 protocol).

Three different doses (5, 25 and 50 nmol /4 × 10^4^ cells) of a control miRNA mimic were evaluated to find the optimal dose to transfect MSCs (Figure 2B). The dose 25 nmol/4 × 10^4^ cells was selected for subsequent experiments. The transfection efficiency was evaluated by qRT-PCR with the miScript PCR system (Qiagen, Venlo, The Netherlands), following the manufacturer’s protocol.

These electroporation conditions did not affect cell viability (not shown).

Selected miRNA mimics (hsa-miR-10a-5p, hsa-miR-29a-3p, hsa-miR-127-3p, hsa-miR-486-5p) (Qiagen) were used to enrich MSCs (600 pmol/10^6^ cells). As a control, MSCs were transfected with AllStars Negative Control siRNA (SCR-Qiagen) and used for normalization of transfection with different mimics. For modified MSCs, EV collection was carried out starting from a four sub-confluent flask (T75, Euroclone S.p.A) 24 h after the electroporation, starving MSCs over-night in RPMI (Euroclone). The collected medium was centrifuged at 2000 *g* for 20 min to eliminate cell debris. Supernatant was then micro-filtrated and concentrated by an Amicon^®^ Ultra 15 mL 3 kDa cut off filter (Merck-Millipore, Darnstadt, Germany) at 4000 rpm 60 min at 4 °C. EV samples were stored at −80 °C, with addition of 1% DMSO (Sigma).

### 4.3. EV Characterization

Analysis of size distribution and enumeration of EVs from naïve MSCs and from MSC-EP (enriched or not with specific miRNA mimics) were performed using NanoSight NS300 (NanoSight Ltd., Amesbury, UK) equipped with a 405 nm laser and nanoparticle tracking analysis (NTA) 3.2 software, as described in Reference [6]. Using a laser light source, particles in the sample are illuminated and the scattered light is captured by the camera and displayed on a connected computer running NTA. Using NTA, the particles are automatically tracked and sized based on Brownian motion and the diffusion coefficient. Three videos of 30 s were recorded to perform the analyses.

EV-surface expression markers were analyzed by Guava easyCyte™ Flow Cytometer (Millipore), as previously described [21]. FITC-, PE- or APC-conjugated antibodies against CD44, CD29, CD73 and CD63 (all from Miltenyi Biotec GmbH, Bergisch Gladbach, Germany) were employed. Isotopic IgG was used as the negative control. Briefly, EVs were incubated at 4 °C for 15 min with the antibodies, then diluted 1:3 and acquired immediately. Samples were acquired using a Guava easyCyte Flow Cytometer (Millipore) and analyzed with InCyte software.

Transmission electron microscopy was performed on CTRL-EVs and EV-EP placed on 200 mesh nickel formvar carbon-coated grids (Electron Microscopy Science, Hatfield, PA, USA) and left to adhere for 20 min, as described in Reference [22]. The grids were then incubated with 2.5% glutaraldehyde containing 2% sucrose and, after washings in distilled water, the EVs were negatively stained with NanoVan (Nanoprobes, Yaphank, NK, USA) and observed using a Jeol JEM 1010 electron microscope (Jeol, Tokyo, Japan).

### 4.4. RNA Analysis

RNA from MSC-EP was extracted by TRIzol™ (Ambion, Thermo Fisher Scientific, Waltham, MA, USA) according to the manufacturer’s protocol.

Only for RNA analysis, Exoquick (System Biosciences, LLC, Palo Alto, CA, USA) was used to precipitate EVs obtained from MSC-EP (EV-EP), MSC transfected with scrambled siRNA (EV-SCR) or with the selected mimics (EV-miR127, EV-miR10a, EV-miR486, EV-miR29a). RNA was extracted with RNA/DNA/Protein Purification Plus Kit (Norgen Biotek Corp, Thorold, ON, Canada), following the manufacturer’s protocol.

RNA concentration was spectroscopically determined by NanoDrop2000 (Thermo Fisher Scientific). cDNA was synthetized and RT-PCR was performed by using miRCURY™ LNA™ Universal RT microRNA PCR (Exiqon-Qiagen, Vedbaek, Denmark). Specific primers set for hsa-miR-127-3p, hsa-miR-10a-5p, hsa-miR-29a-3p, hsa-miR-486-5 were used. U6 spike-in was used for housekeeping (Exiqon-Qiagen). Data were normalized with respect to MSC transfected with the SCR and were represented as relative quantification (RQ) ± SEM.

### 4.5. Integrating miRNA Expression in MSC EVs and RNA Analysis in AKI Animals

To identify potentially relevant miRNAs carried by MSC EVs and involved in the positive readout associated with EVs treatment, we proceeded as follows: For each miRNA family listed in TargetScan we generated a list of predicted targets. We then compared the fold-change in expression of these targets with the fold-change in expression of all the genes that were predicted targets of miRNAs but not of the miRNA families under analysis. The miRNA families selected were those for which the targets showed significant down-regulation in the kidneys of AKI mice treated with MSC-derived EVs vs. untreated AKI mice (AKI), as detected in Reference [12]. The obtained miRNA families were then matched with the list of miRNAs detected inside the total extracellular vesicle population (100K TOT) in Reference [6].

### 4.6. mTEC Proliferation Assay

mTEC were seeded in 96-well plates at a density of 1000 cells/well and maintained in a hypoxia chamber (Stem Cell Technology, Vancouver)) for 48 h with 1% O_2_ in DMEM + 10% FCS (positive control), DMEM + 0% FCS (negative control). During the reoxygenation step, mTEC were treated for 24 h with mimics of selected miRNAs (100 nM) or 500 EV/mTEC. Cell proliferation was assessed by a 5-bromo-2′-deoxy-uridine (BrdU) incorporation assay (Roche Applied Science, Mannheim, Germany).

### 4.7. SCID Mouse Model of AKI

Animal studies were conducted in accordance with the National Institutes of Health Guide for the Care and Use of Laboratory Animals. All procedures were approved by the Italian Health Ministry (authorization number: 211/2016-PR).

AKI was induced by an intramuscular injection (IM) of glycerol (Sigma) in SCID mice, as described previously [6,20]. Male SCID mice were anesthetized with an IM injection of zolazepam (80 mg/kg) and xilazina (16 mg/kg) and then injected with 8 mL/kg of 50% glycerol in water. Half the dose was injected into each muscle of the inferior hind limbs. Three days after the glycerol injection, the mice were treated intravenously with either 120 μL of the vehicle alone (*n* = 7) or containing 165 × 10^6^ or 82.5 × 10^6^ particles of EVs derived from control MSCs (EV-CTRL, *n* = 8), from MSC-EP (EV-EP, *n* = 8/doses), or from EP-MSC enriched with specific miRNA mimics (EV-miR127, EV-miR486, EV-miR10a, *n* = 6/group/doses). The mice were sacrificed 5 days after the glycerol injection (2 days after EV treatment).

Blood samples were collected 5 days after glycerol-induced AKI for the measurement of blood urea nitrogen (BUN) and creatinine. BUN was measured by direct quantification of serum urea with a colorimetric assay kit according to the manufacturer’s protocol (Arbor Assays, Ann Arbor, MI, USA). Serum creatinine was measured using a colorimetric microplate assay based on the kinetic Jaffe reaction as per the manufacturer’s protocol (Quantichrom Creatinine Assay; BioAssay Systems, Hayward, CA, USA).

Renal morphology was evaluated through formalin-fixed paraffin-embedded tissue staining, as previously described [6,20]. Briefly, 5-μm-thick paraffin kidney sections were routinely stained with hematoxylin and eosin (Merck-Millipore) for microscopic evaluation. Luminal hyaline casts and cell necrosis (denudation of the tubular basement membrane) were assessed in non-overlapping fields (10 for each section) using a 40× objective (high-power field [HPF]). The number of casts and tubular profiles showing necrosis were recorded in a single-blind manner.

### 4.8. Statistical Analyses

Data were analyzed using the GraphPad Prism 6.0 program. Statistical analysis was performed by employing Student’s *t*-tests, analysis of variance (ANOVA) with Dunnett’s multi-comparison tests or Multiple t test with Helm–Sidak method correction as deemed appropriate. A *p*-value of <0.05 was considered significant. For the bioinformatic analyses, *p* values were generated by Mann–Whitney statistical test comparing the median fold change of genes down-regulated in EV treated animals (Log EV treated vs. AKI untreated) that were target of miRNAs with the non-target genes. Nine miRNA families (Table 1) achieved nominal significance (*p* < 0.05).

## Figures and Tables

**Table 1:** List of miRNA families for which targets were significantly modulated by extracellular vesicle (EV) administration in acute kidney injury (AKI) mice.

	FC Non-Targets	FC Targets	*p*	Rank
miR-10abc/10a-5p	0.03	0.06	0.0313	1
let-7/98/4458/4500	0.03	0.06	0.0476	2
miR-127/127-3p	0.03	0.25	0.0431	12
miR-30abcdef/30abe-5p/384-5p	0.03	0.07	0.0003	13
miR-29abcd	0.03	0.07	0.0047	24
miR-192/215	0.03	0.14	0.0035	50
miR-140/140-5p/876-3p/1244	0.03	0.10	0.0177	72
miR-377	0.03	0.08	0.0203	96
miR-202-3p	0.03	0.08	0.0060	124

miRNAs with targets that showed significant down-regulation in EV treated-vs. untreated-AKI mice were listed and correlated with their enrichment in EVs isolated at 100,000 *g* (*p* value < 0.05). Non-targets: Median fold change (FC, logarithmic treated vs. untreated) of the genes that are not miRNA targets. Targets: Median FC of the genes that are miRNA targets. *p*: *p*-value generated by Mann–Whitney test, comparing the FC of targets vs. non-target miRNAs. Rank: List of the expressed miRNAs based on their abundance inside EVs.

**Table 2:** Electroporation protocols tested to transfect MSCs.

	EP1	EP2	EP3
Voltage	990	1100	990
msec	40	30	30
Pulse	1	1	2
